# Letter from the New Editor in Chief

**DOI:** 10.1017/S0016672319000028

**Published:** 2019-02-12

**Authors:** Marc Tischkowitz

**Affiliations:** Department of Medical Genetics, University of Cambridge

When I first got the email invite for the Editorship of *Genetics Research* my instinct was to dismiss it outright. It was not a journal I was familiar with, could it even have been just another of the spam emails from predatory/fake journals we are all blighted with each day? But then I took a closer look. Granted, the impact factor was low, but it has a long and established history, having been founded in 1960 as *Genetical Research*. Moreover it comes from the Cambridge University Press stable, the oldest and one of the most venerated publishing houses which, importantly, is a non-commercial enterprise with the highest of ethical standards. Perhaps there was something more to this journal and my initial impression was mistaken? I decided to meet the editorial team and was impressed by the professionalism, enthusiasm and drive to give this publication a new life as an open access journal. I was quickly convinced that there is real potential to develop the journal into something special that could make a significant contribution to the scientific community. I agreed to take on the position and started at the beginning of this year. As a clinician-scientist with over 20 years experience of publishing papers I am very aware of the trials and tribulations that authors face when submitting their work to journals. I have had many excellent experiences but also some downright awful ones, and I am keen to use my knowledge to bring out the best in the journal and the service it provides to the scientific community. We are all aware that Impact Factors are a flawed metric but in the absence of anything better we continue to abide by the system. GR currently has a low IF and I realise it may well not be your first choice journal, but we will do our best to provide an excellent service with fast turnaround times and clear communication with submitting authors. We are ambitious and with the new impetus from Open Access we are confident that we can move the journal up the rankings over the next few years.

GR was first published in 1960, only a few years after discovery of the double helix and the correct determination of the human diploid chromosome. Back then it was mainly focussed on mouse, drosophila and other non-human genetics and published works by world-renowned scientists such as Mary Lyon, Charlotte Auerbach and John Maynard Smith (who is also the author of GR's most cited paper, entitled “*The hitch-hiking effect of a favourable gene*” and, published in 1974). How can we bring such an illustrious journal back to its former glory given the modern day challenges of working in a crowded and highly competitive field? We have taken the first steps by making it Open Access and appointing a new Editorial Board. Our aim is to publish high quality research in all areas of genetics and to better serve the community we will solicit reviews and opinion pieces from leaders in diverse fields of genetics. We welcome methods papers and novel findings that may not be fully tested but could potentially be ground-breaking. We will give speedy consideration to articles that have been reviewed and rejected by higher impact factor journals and give a quick decision on their suitability for publication in GR. We will encourage submissions from up and coming scientists as well as established ones. We will engage with social media to publicise new articles and give them maximum exposure by capitalising on the extensive resources available to us through the Press. Our aim is to stay closely connected with the genetics community and serve you to the best of our abilities.

We have seen some dramatic changes since the inception of GR and today's world of research bears very little resemblance to that seemingly cosy and quaint time of early discovery in the last century. Even ten years ago it was hard to conceive that whole genome sequencing would be a viable tool for a modestly funded researcher. The huge amounts of data generated from high throughput genetic analysis present new challenges not just in analysis and interpretation but also in the ethical, social and legal implications and we welcome research articles and opinion pieces on all these areas. In fact we have recently broadened the scope of the journal to “all aspects of genetics, or in any field which has an important bearing in genetics”. Next year will be the 60^th^ anniversary for GR and we will celebrate this with a series of specially commissioned pieces looking back over the last six decades and forward to the future. In the meantime please do get in touch with your suggestions for articles and tell us how you think we can best serve the scientific community and beyond with our new Open Access platform. We are genuinely interested in hearing your ideas and feedback so please do contact us at geneticsresearch@cambridge.org

Finally, I would like to thank my predecessor Professor Noam Shomron and his Editorial Board for their hard work in running the Journal over the last six years. I look forward to working with our new team to build on this foundation and make *Genetics Research* a truly excellent journal that authors will be proud to publish in.

